# In‐Cell Activation of Organo‐Osmium(II) Anticancer Complexes

**DOI:** 10.1002/anie.201610290

**Published:** 2016-12-21

**Authors:** Russell J. Needham, Carlos Sanchez‐Cano, Xin Zhang, Isolda Romero‐Canelón, Abraha Habtemariam, Margaret S. Cooper, Levente Meszaros, Guy J. Clarkson, Philip J. Blower, Peter J. Sadler

**Affiliations:** ^1^ Department of ChemistryUniversity of WarwickCoventryCV4 7ALUK; ^2^ Division of Imaging Sciences and Biomedical EngineeringKing's College LondonSt. Thomas HospitalLondonSE1 7EHUK

**Keywords:** anticancer agents, bioinorganic chemistry, glutathione, metallodrugs, organo-osmium complexes

## Abstract

The family of iodido Os^II^ arene phenylazopyridine complexes [Os(η^6^‐p‐cym)(5‐R^1^‐pyridylazo‐4‐R^2^‐phenyl))I]^+^ (where p‐cym=para‐cymene) exhibit potent sub‐micromolar antiproliferative activity towards human cancer cells and are active in vivo. Their chemical behavior is distinct from that of cisplatin: they do not readily hydrolyze, nor bind to DNA bases. We report here a mechanism by which they are activated in cancer cells, involving release of the I^−^ ligand in the presence of glutathione (GSH). The X‐ray crystal structures of two active complexes are reported, **1**‐I (R^1^=OEt, R^2^=H) and **2**‐I (R^1^=H, R^2^=NMe_2_). They were labelled with the radionuclide ^131^I (β^−^/γ emitter, t_1/2_ 8.02 d), and their activity in MCF‐7 human breast cancer cells was studied. **1**‐[^131^I] and **2**‐[^131^I] exhibit good stability in both phosphate‐buffered saline and blood serum. In contrast, once taken up by MCF‐7 cells, the iodide ligand is rapidly pumped out. Intriguingly, GSH catalyzes their hydrolysis. The resulting hydroxido complexes can form thiolato and sulfenato adducts with GSH, and react with H_2_O_2_ generating hydroxyl radicals. These findings shed new light on the mechanism of action of these organo‐osmium complexes.

Organometallic complexes show promise as a new generation of anticancer drugs. These include cyclopentadienyl complexes of Fe^II^, Rh^III^ and Ir^III^, and arene complexes of Ru^II^, Os^II^, Rh^III^ and Ir^III^.^1^ They offer the prospect of mechanisms of action that differ from Pt^II^ complexes, which are currently the most widely‐used drugs in the clinic. Organometallic drugs have the potential to expand the range of treatable cancers, cause fewer side‐effects, and provide activity against Pt‐resistance, a current clinical problem. Os^II^ arene complexes containing a chelated phenylazopyridine ligand, [Os(*η* ^6^‐*p*‐cym)(5‐R^1^‐pyridylazo‐4‐R^2^‐phenyl))I]^+^, possess typical half‐sandwich “piano‐stool” structures. The X‐ray crystal structures of complexes **1**‐I**⋅**PF_6_⋅0.5 EtOH (R^1^=OEt, R^2^=H) and **2**‐I**⋅**PF_6_ (R^1^=H, R^2^=NMe_2_) show long Os−I bonds (2.6974(2) and 2.7083(2) Å, respectively), and relatively flat *N*,*N*‐chelated phenylazopyridine ligands (Figure 1). Unlike cisplatin, **1**‐I and **2**‐I are relatively inert. They do not readily hydrolyze nor bind to DNA in vitro.^2^ However, **1**‐I and **2**‐I exhibit activity that is equal to or greater than that of the clinical drug cisplatin in a wide range of cancer cell lines, despite having a contrasting profile of chemical reactivity (Table 1), and are capable of overcoming resistance to clinical platinum drugs.^2^ Complex **2**‐I is 49× more potent than cisplatin in a panel of 809 cancer cell lines,^3^ and active in vivo.^4^ We have shown that this drug‐candidate is capable of modulating the cellular redox balance, increasing dramatically the production of reactive oxygen species (ROS) in cancer cells, and has enhanced potency when used in combination with low doses of l‐buthionine sulfoximine (l‐BSO), which depletes cellular levels of glutathione (GSH, γ‐l‐Glu‐l‐Cys‐Gly).^5^ GSH is an important cellular antioxidant that plays a key role in the detoxification of ROS^6^ and platinum drugs.^7^ The organo‐Ru analogue of **2**‐I binds to GSH and catalyzes its oxidation to GSSG, probably through redox mediation by the azo group.^8^ Furthermore, the Ru–SG thiolate adduct formed from chlorido Ru^II^ arene ethylenediamine complexes can be oxidized to Ru‐SOG sulfenate species in the presence of O_2_; these reactive adducts facilitate the interaction between the Ru complexes and guanine/DNA.^9^ Other Ru complexes can also be activated by GSH.^10^ Additionally, the coupling of anticancer activity to redox reactions of metals and ligands in organometallic complexes provides intriguing possibilities for novel mechanisms of action, as is illustrated especially by the ferrociphen series of complexes.^11^ We show here that, surprisingly, the antioxidant GSH not only promotes activation of iodido Os^II^ arene azopyridine complexes through aquation, but also forms sulfenate adducts.

**Figure 1:**
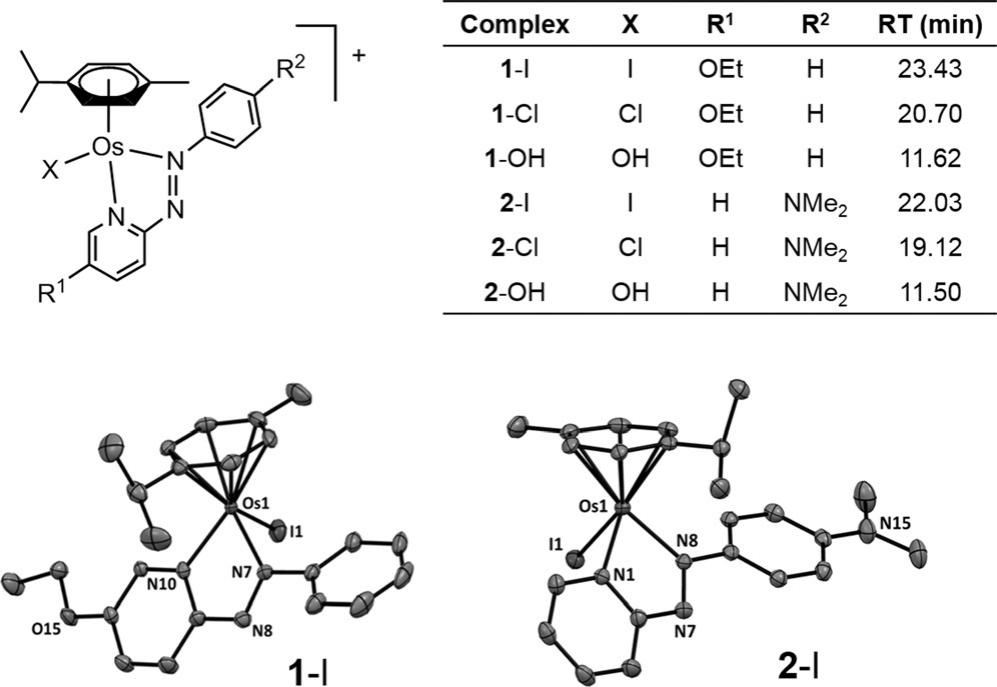
Complexes studied here, HPLC retention times, and ORTEP diagrams for complexes **1**‐I and **2**‐I. Ellipsoids are shown at the 50 % probability level. H atoms, counter ions and solvent molecules are omitted for clarity.

**Table 1:** Antiproliferative activity of complexes **1** and **2** towards A2780 human ovarian and MCF‐7 breast cancer cells.

Complex	IC_50_ [μm]	
	A2780	MCF‐7
**1**‐I	0.92±0.02	1.2±0.2
**1**‐Cl	15.1±0.5	n.d.
**1**‐OH	0.27±0.02	14.3±0.3
**2**‐I	0.15±0.02^[a]^	0.20±0.01^[a]^
**2**‐Cl	1.8±0.3^[a]^	1.1±0.8^[a]^
cisplatin	1.2±0.2	7.4±0.2

[a] Ref. 2.

Initially, we used the β^−^/γ emitter ^131^I (*t*_1/2_ 8.02 d) as a radiotracer to label **1**‐I and **2**‐I and study their cell uptake and efflux. Complexes **1**‐[^131^I] and **2**‐[^131^I] were synthesized by exchange of Cl^−^ in **1**‐Cl and **2**‐Cl (Figure 1), by reacting a large excess of complex with Na^131^I. The exchange was complete in 2 h for **1**‐Cl, but required 18 h for **2**‐Cl. Reactions were followed by radio‐TLC chromatograms (Figure S1 in the Supporting Information), and reverse‐phase HPLC with simultaneous detection at 254 nm and γ‐emission (Figure S2). Os‐OH, Os‐Cl and Os‐I species were identified using LC‐MS, and the formation of Os‐^131^I was confirmed by performing the radio‐labelling in presence of 0.5 mol equiv of cold NaI (Figures 1 and S2). Complexes **1**‐[^131^I] and **2**‐[^131^I] were purified via preparative HPLC to remove residual chlorido complex, diluted in PBS to pH 6–7, then frozen immediately and stored at 193 K to minimize decomposition until required.

Both **1**‐[^131^I] and **2**‐[^131^I] exhibited good stability after 24 h at 310 K in human blood serum (ca. 25 %, and 11 % iodide released, respectively), and cell culture medium (ca. 27 %, and 14 %, Figure S3). However, when MCF‐7 breast cancer cells were treated with the tracer‐complexes, we observed a rapid release of free ^131^I into the supernatant. After 24 h, 97 % and 99 % of **1**‐[^131^I] and **2**‐[^131^I], respectively, lost the radioactive iodide ligand, the rate of loss being greater for **2**‐[^131^I] (Figure 2). Interestingly, the maximum amount of intracellular ^131^I was observed after only 5 min for both **1‐**[^131^I] and **2**‐[^131^I] (0.8 % and 1.8 % of total ^131^I/10^6^ cells, respectively). After this time, the level of accumulated ^131^I declined steadily (Figure 3 and S4).

**Figure 2:**
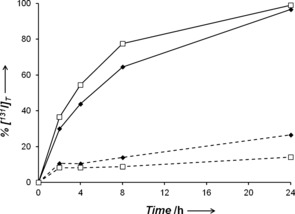
Release of free ^131^I into the supernatant of cell culture medium at various times after incubation with **1**‐[^131^I] (♦) or **2**‐[^131^I] (□), in the absence (dashed lines) or presence (solid lines) of MCF‐7 breast cancer cells. Percentages were determined by HPLC peak integrals.

**Figure 3:**
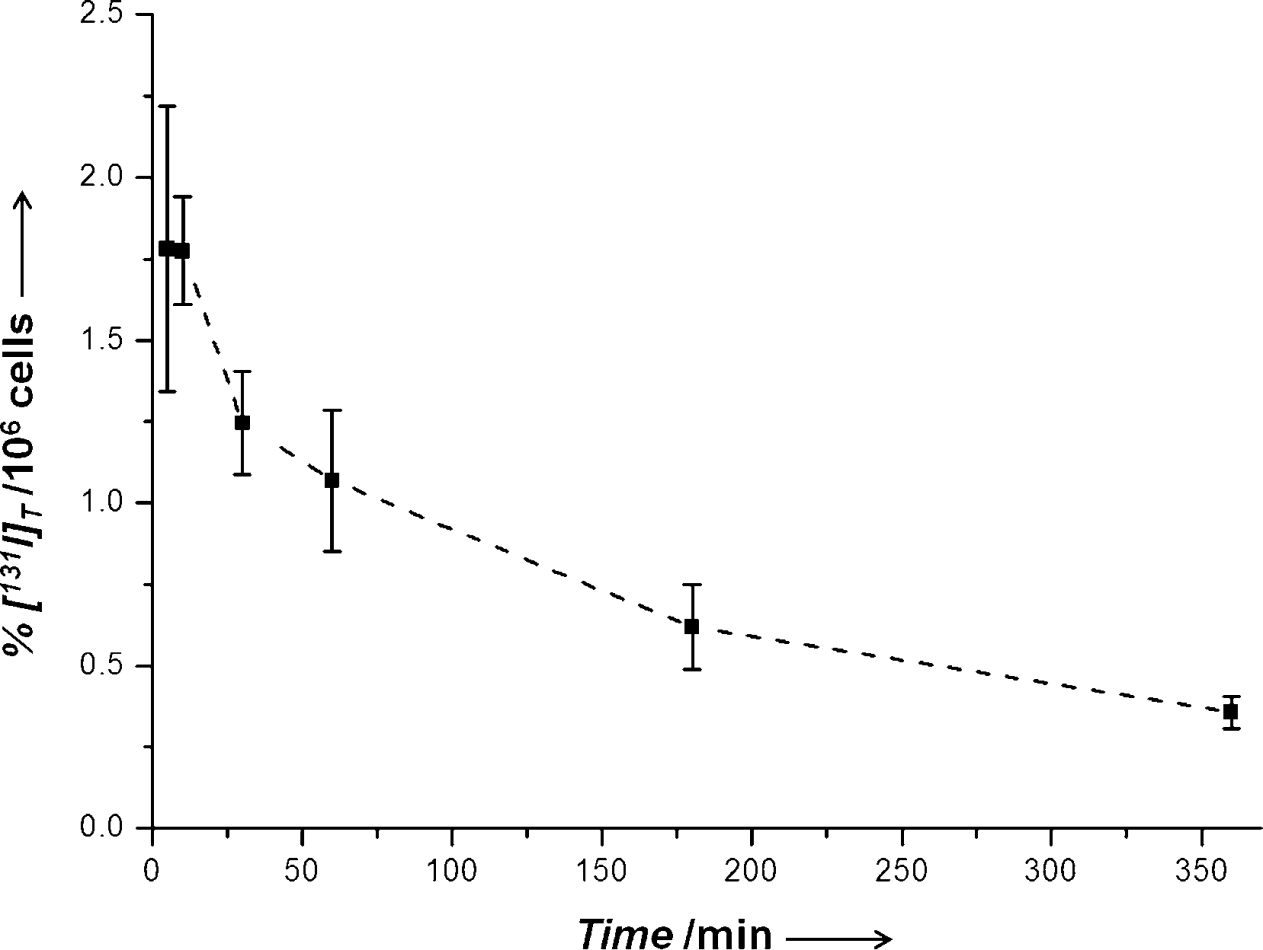
Cellular accumulation of ^131^I in MCF‐7 breast cancer cells at various times after incubation with **2**‐[^131^I].

We have shown previously that significant amounts of complex **2**‐I are taken up by cancer cells within the first 30 min and the amount of Os accumulated increases with longer incubation times.^12^ Thus the free iodide detected here might arise from the efflux of ^131^I, perhaps via chloride transport channels.^13^ In general, iodide transport mechanisms appear to be little studied apart from cells in the thyroid. However, complex **1**‐I was found not to hydrolyze readily under either intracellular (23 mm) or intranuclear (4 mm) levels of chloride (Figure S5), and therefore the observed loss of the iodido ligand might arise from interaction with intracellular biomolecules.

These surprising results led us to study the reactions between **1**‐I or **2**‐I (75 μm) and GSH (1 mol. equiv) using HPLC. Surprisingly, these experiments showed that the hydrolysis of the Os−I bond is promoted by GSH: a new set of peaks identified as **1**‐OH and **2**‐OH by LC‐MS began to appear after 3 h of incubation and no GSH adducts were detected. The extent of hydrolysis was greater for **1**‐I (71 %) than for **2**‐I (33 %) after 24 h (Figure 4 and S7). In contrast, less than 1 % of **1**‐OH and **2**‐OH was observed when **1**‐I and **2**‐I were incubated under the same conditions in the absence of GSH (Figure S8), indicating that hydrolysis is induced by the presence of the thiol‐containing tripeptide. The p*K*_a_ of **1**‐OH_2_ was determined by NMR titration to be 4.55±0.01, indicating that the more stable Os‐OH species will predominate at physiological pH values (ca. 7.4) over the more labile Os‐OH_2_ species (Figure S9). Furthermore, incubation of **1**‐I or **2**‐I with a large excess of GSH (100 mol. equiv) accelerated their hydrolysis rates dramatically (complete in 3 h for **1**‐I and 6 h for **2**‐I; Figures 4 and S7). Moreover, the formation of thiolato (GS^−^) and sulfenato (GSO^−^) adducts of **1**‐I and **2**‐I were observed (Figures 4 and S7), and confirmed by LC‐MS (Figure S10 and Table S1). This appears to be the first report of Os^II^–sulfenato adducts. Similar adducts were observed when **1**‐OH was incubated with just 1 mol. equiv of GSH, suggesting that hydrolysis of the Os−I bond is essential for GSH binding to **1**‐I and **2**‐I. Complexes **1**‐Cl and **2**‐Cl behaved in a similar way, but showed faster rates of hydrolysis and GSH binding, indicating greater reactivity than their Os‐I analogues (Figure S11). Similar results were observed when 1 mol. equiv *N*‐acetyl‐l‐cysteine (NAC) or ascorbic acid (also reducing agents) were used instead of GSH (Figures S12 and S13). Interestingly, the presence of 30 %v/v acetone almost completely hindered the reaction of **2**‐I with NAC and hydrolysis of **2**‐I (Figure S14), accounting for our previous observation of a lack of reaction between **2**‐I and NAC.^2^

**Figure 4:**
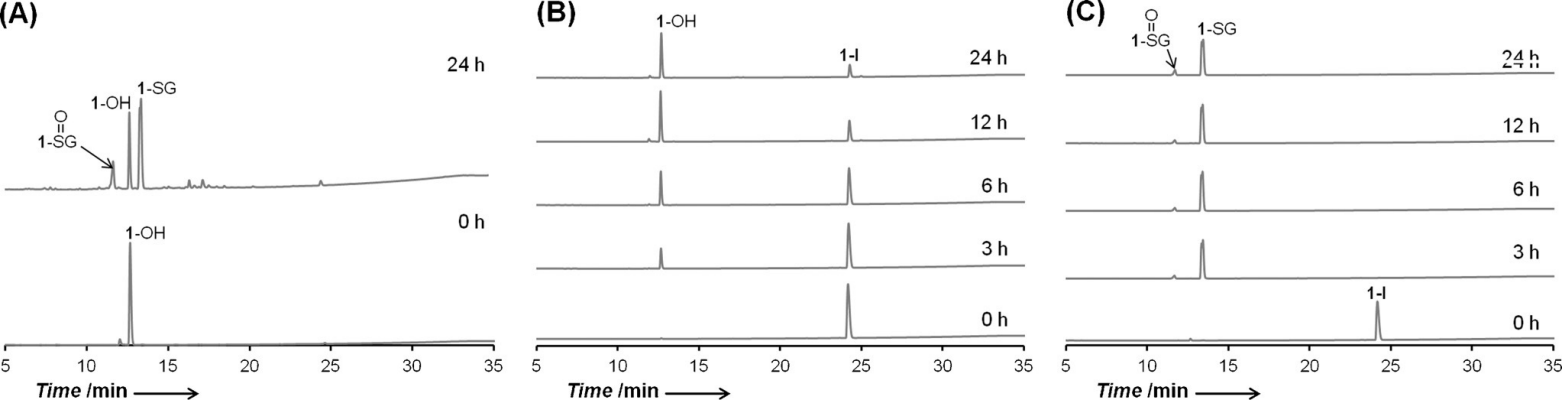
HPLC separation of products from reactions of complexes with GSH in 7.5 mm phosphate buffer (pH 7.4) at 310 K for various times. A) 1 mm **1**‐OH with 1 mol. equiv GSH in 0.1 m phosphate buffer. B) 75 μm **1**‐I with 1 mol equiv GSH, and C) 75 μm **1**‐I with 100 mol equiv GSH.

GSH‐mediated hydrolysis of **1**‐I and **2**‐I can therefore generate Os species that are more reactive than the parent iodido complexes. When the reaction of **2**‐I (75 μm) and GSH (0.08–7.5 mm) was repeated in the presence of an intracellular concentration of NaCl (25 mm), a new peak corresponding to **2**‐Cl was observed after 24 h incubation with GSH concentrations up to ca. 2 mm. Incubations at higher GSH concentrations led to the formation of **2**‐OH, thiolato and sulfenato adducts only (Figure S15).

Complexes **1**‐I and **2**‐I and their Cl and hydroxido analogues react with H_2_O_2_, a ROS overproduced in cancer cells, to generate hydroxyl radicals detected by EPR spectroscopy using the spin‐trap 5‐diethoxyphosphoryl‐5‐methyl‐1‐pyrroline *N*‐oxide (DEPMPO, Figures 5 and S16). The efficiency of OH^**.**^ radical generation followed the trend Os‐OH > Os‐Cl > Os‐I. This is the same reactivity trend observed for reactions with GSH, and suggests that intracellular hydrolysis of **1**‐I and **2**‐I might be a key activation step for their biological activity. Additionally, the low reactivity of iodido complexes **1**‐I and **2**‐I compared to **1**‐Cl and **2**‐Cl might explain why chlorido derivatives are 10× less active in inhibiting the proliferation of cancer cells than their iodido analogues (Table 1). The chlorido complexes might undergo side‐reactions more readily, leading to detoxification before reaching target sites. Interestingly, **1**‐OH is more active than **1**‐I in A2780 but not MCF‐7 cells. This might be due to higher levels of GSH in MCF‐7 compared to A2780 cells (ca. 40 versus 30 nmol GSH/mg^−1^ protein, respectively)^15^ but other factors may also play a role.

**Figure 5:**
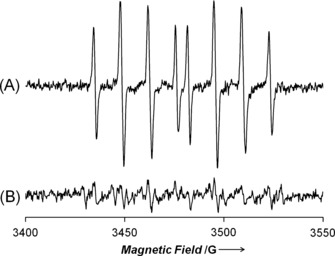
X‐band EPR spectra showing A) trapping of OH radicals by the spin‐trap DEPMPO (6 mm) from reaction of H_2_O_2_ (10 mm) with **1**‐OH (1 mm) in 75 mm phosphate buffer, pH 7.4, and B) quenching in presence of ethanol (10 mm). The EPR parameters of the trapped radical are typical of trapped HO**^.^** (*g*: 2.01, *a* ^N^_NO_: 14.02 G, *a* ^P^: 47.01 G, *a* ^H^_β_: 13.22 G).^14^

In conclusion, we show that, in contrast to platinum anticancer drugs, GSH can provide a route for intracellular activation of osmium prodrugs **1**‐I and **2**‐I (Figure S17). This involves hydrolysis of the Os−I bond, most probably via redox mediation by the azo group and transformation into more reactive hydroxido forms **1**‐OH and **2**‐OH, releasing the I^−^ ions from the cell in the process. Such Os‐OH complexes can bind to Cl^−^ ions or GSH, forming chlorido, thiolato and also Os‐sulfenato adducts, and can also catabolize H_2_O_2_ generating OH^**.**^ radicals. The facile ability of GSH to form sulfenato adducts (compared to NAC) was especially notable. Organo‐osmium complexes can cause dramatic changes in the redox state of cancer cells^5^ and these new insights into their activation mechanism pave the way for furthering our understanding of their target sites, which might involve attack by osmium on proteins in the cytoplasm and mitochondria.

## Experimental Section

Synthesis of **1**‐I⋅PF_6_: [Os(η^6^‐*p*‐cym)I_2_]_2_ (100 mg, 86.5 μmol) was dissolved in ethanol (10 mL), and a solution of 2‐(phenylazo)‐5‐ethoxypyridine (41.3 mg, 181.6 μmol) in ethanol (5 mL) was added drop‐wise. The mixture was stirred for 18 h at ambient temperature, and ammonium hexafluorophosphate (140.9 mg, 0.86 mmol) was added. The mixture was concentrated under reduced pressure to ca. 3 mL and placed in a freezer overnight. The dark crystalline precipitate was collected via vacuum filtration, washed with ice‐cold ethanol (2×1 mL) and diethyl ether (2×5 mL), and dried overnight in a vacuum desiccator. Yield: 119.6 mg (84 %). ^1^H NMR (CD_3_OD): *δ*=9.07 (d, 1 H, *J*=2.6 Hz), 8.84 (d, 1 H, *J*=9.1 Hz), 8.04–8.01 (m, 2 H), 7.94 (dd, 1 H, *J*=9.1, 2.6 Hz), 7.73–7.63 (m, 3 H), 6.47–6.46 (m, 1 H), 6.16–6.15 (m, 1 H), 6.03–6.02 (m, 1 H), 5.96–5.95 (m, 1 H), 4.50–4.38 (m, 2 H), 2.70 (s, 3 H), 2.45 (sept., 1 H, *J*=6.9 Hz), 1.55 (t, 3 H, *J*=7.0 Hz), 0.94–0.92 ppm (2x d, 6 H, *J*=6.9 Hz). ESI‐MS calculated for C_23_H_27_IN_3_OOs^+^: *m*/*z* 680.1. Found: 679.9. CHN analysis: Found: C, 33.30 %; H, 3.26 %; N, 4.98 %. Calculated for C_23_H_27_F_6_IN_3_OOsP: C, 33.54 %; H, 3.30 %; N, 5.10 %.

Radiolabelling: 50 μL of **1**‐Cl⋅PF_6_ or **2**‐Cl⋅PF_6_ in methanol (5 mg mL^−1^) was transferred into a 2 mL plastic sealable tube and combined with Na^131^I (30–70 MBq) in 0.1 mm NaOH solution. Further water was added to give a water:methanol (1:1, *v*/*v*) solvent matrix, then the mixture was heated for 18 h at 333 K with 300 rpm stirring. The radio‐labelled complexes were purified by preparative radio‐HPLC and diluted with three parts phosphate buffered saline (PBS) and stored at 193 K.

## Conflict of interest

The authors declare no conflict of interest.

## Supporting information

As a service to our authors and readers, this journal provides supporting information supplied by the authors. Such materials are peer reviewed and may be re‐organized for online delivery, but are not copy‐edited or typeset. Technical support issues arising from supporting information (other than missing files) should be addressed to the authors.

Supplementary
