# Supplementary material for: In‐Cell Activation of Organo‐Osmium(II) Anticancer Complexes
Source: Angew Chem Int Ed Engl. 2016 Dec 21;56(4):1017–20. doi: 10.1002/anie.201610290 (PMC5412917; doi:10.1002/anie.201610290)
Supplement: Supplementary file 1 — Supplementary [file ANIE-56-1017-s001.pdf]

Angewandte Chemie
Eine Zeitschrift der Gesellschaft Deutscher Chemiker

Supporting Information

In-Cell Activation of Organo-Osmium(II) Anticancer Complexes

*Russell J. Needham+, Carlos Sanchez-Cano+, Xin Zhang, Isolda Romero-Canelón, Abraha Habtemariam, Margaret S. Cooper, Levente Meszaros, Guy J. Clarkson, Philip J. Blower, and Peter J. Sadler**

anie_201610290_sm_miscellaneous_information.pdf

Supporting Information

Table of Contents

	Page
Experimental	S2
Table S1	S10
Figures S1- S17	S10 - S22

Experimental

Materials. Osmium dimers ([Os(η^6-*p*-cym)X_2]$_2$, where X= Cl or I) were prepared following previously reported literature procedures.[1,2] 2-Amino-5-fluoropyridine, nitrosobenzene, silver nitrate, hydrogen peroxide 30 wt.%, perchloric acid 70 wt.%, reduced glutathione, N-acetyl-*L*-cysteine and *L*-ascorbic acid were all purchased from Sigma Aldrich. Sodium hydroxide was purchased from Fisher Scientific. All other solvents and reagents for synthesis and analysis were purchased from commercial suppliers and used as received. EPR tubes were purchased from Wilmad Labglass, and the spin trap 5-(diethoxyphosphoryl)-5-methyl-1-pyrroline-N-oxide (DEPMPO) was obtained from Enzo Life Sciences.

Iodine-131 was purchased from Perkin-Elmer as an aqueous sodium iodide-131 solution (74 MBq; 185 GBq/mg) in 0.1 M NaOH (pH 12-14). Human blood serum (male, O-positive) was obtained from a volunteer at the St. Thomas's Hospital blood centre facility. Blood was drawn into 2x 5 mL BD vacutainer tubes with BD Hemogard closures. The tubes contained a clot activator and a gel for serum separation. The blood clotted after ~30 min and the serum was separated by centrifugation (3600 rpm, 10 min). Approximately 5 mL of serum was obtained and stored in a refrigerator for no more than 4 days. D-MEM cell culture medium, RPMI-1640 cell culture medium, penicillin/streptomycin mixture, foetal bovine serum, *L*-glutamine, phosphate buffered saline solution (PBS), trypsin and propidium iodide were all purchased from Sigma-Aldrich.

Stock phosphate buffer solutions (600 mM) were prepared by dissolving monosodium phosphate monohydrate (1.56 g, 11.3 mmol) and disodium phosphate heptahydrate (13.1 g, 48.9 mmol) (both from Fisher Scientific) in doubly deionised water (100 mL). The pH of the phosphate buffer solution was adjusted to pH 7.4(±0.1) using sodium hydroxide. Phosphate-buffered D_2O solutions were prepared in a similar manner, adjusting the pH* to 7.4(±0.1) using sodium hydroxide in D_2O without correcting for the effect of deuterium on the glass electrode.

Synthesis of 2-(phenylazo)-5-fluoropyridine (5-F-AZPY). 2-Amino-5-fluoropyridine (1.00 g, 8.92 mmol) was stirred in a 1:4 mixture of benzene (5 mL) and water (20 mL). Sodium hydroxide (4.28 g, 0.11 mol) was added and the mixture was left to stir for 10

min at 323 K under N_2. Nitrosobenzene (0.96 g, 8.92 mmol) was added to the stirred mixture and an instant colour change to green was observed. The mixture was heated at 373 K overnight under N_2, and turned dark red-brown. The layers were separated and the aqueous layer was extracted with toluene (3 x 50 mL). The combined organic extracts were washed with water (3 x 30 mL), dried over $MgSO_4$ and filtered. The solvent was removed under reduced pressure to produce an oily red precipitate. The precipitate was then dissolved in a minimum amount of dichloromethane and purified via flash column chromatography (SiO_2, dichloromethane, R_f = 0.52). The collected fractions were combined and the solvents were removed under reduced pressure, yielding an orange precipitate. The product was dried overnight in a vacuum desiccator. Yield: 469.1 mg (26%). ^1H NMR ($CDCl_3$): δ 8.52 (d, 1H, J = 2.9), 7.96-7.98 (m, 2H), 7.83 (dd, 1H, J = 8.8, 4.3 Hz), 7.55-7.54 (m, 1H), 7.46-7.49 (m, 3H). ESI-MS calculated for $C_{11}H_8N_3F + H^+$: m/z 202.1. Found: 202.1. CHN analysis: Found: C, 65.39%; H, 3.90%; N, 20.71%. Calculated for $C_{11}H_8N_3F$: C, 65.66%; H, 4.01%; N, 20.88%.

Synthesis of 2-(phenylazo)-5-ethoxypyridine (5-EtO-AZPY). 2-(Phenylazo)-5-fluoropyridine (100.0 mg, 0.497 mmol) was dissolved in ethanol (20 mL) and an aqueous KOH solution was added (5 mol. equiv.). The mixture was heated to reflux for 18 h and the product was extracted with dichloromethane (20 mL) and washed with water (3 x 20 mL). The dichloromethane extract was dried over $MgSO_4$, filtered, and the solvent was removed under reduced pressure. The crude product was re-crystallised from a minimum amount of hot diethyl ether. Orange crystals were collected via vacuum filtration, washed with ice-cold diethyl ether and dried overnight in a vacuum desiccator. Yield: 78.1 mg (69%). ^1H NMR ($CDCl_3$): δ 8.31 (d, 1H, J = 3.0), 7.92-7.95 (m, 2H), 7.79 (d, 1H, J = 8.8 Hz), 7.39-7.47 (m, 3H), 7.27 (dd, 1H, J = 8.8, 3.0 Hz), 4.10 (q, 2H, J = 7.0 Hz), 1.40 (t, 3H, J = 7.0 Hz). ESI-MS calculated for $C_{13}H_{13}N_3O + H^+$: m/z 228.1. Found: 228.0. CHN analysis: Found: C, 65.39%; H, 3.90%; N, 20.71%. Calculated for $C_{11}H_8N_3F$: C, 65.66%; H, 4.01%; N, 20.88%.

Synthesis of [Os(η^6-*p*-cym)(5-EtO-AZPY)I]CF_3SO_3 (1-I·CF_3SO_3). [Os(η^6-*p*-cym)I_2]$_2$ (50 mg, 43.2 μmol) was dissolved in ethanol (10 mL), and a solution of 2-(phenylazo)-5-ethoxypyridine (20.6 mg, 90.8 μmol) in ethanol (5 mL) was added drop-wise. The mixture was stirred for 18 h at ambient temperature, and ammonium triflate (72.3 mg, 0.43 mmol) was added. The mixture was concentrated under reduced pressure to ~3

mL and placed in a freezer overnight. A dark crystalline precipitate formed, which was collected via vacuum filtration and washed with ice-cold ethanol (2 x 1 mL), diethyl ether (2 x 5 mL), and dried overnight in a vacuum desiccator. Yield: 66.8 mg (93%). ^1H NMR (CD_3OD): δ 9.07 (d, 1H, J = 2.6 Hz), 8.84 (d, 1H, J = 9.1 Hz), 8.01-8.05 (m, 2H), 7.94 (dd, 1H, J = 9.1, 2.6 Hz), 7.69-7.74 (m, 1H), 7.63-7.68 (m, 2H), 6.47-6.46 (m, 1H), 6.17-6.16 (m, 1H), 6.03-6.02 (m, 1H), 5.96-5.95 (m, 1H), 4.38-4.51 (m, 2H), 2.71 (s, 3H), 2.45 (sept., 1H, J = 6.9 Hz), 1.55 (t, 3H, J = 7.0 Hz), 0.92-0.94 (2x d, 6H, J = 6.9 Hz). ESI-MS calculated for $C_{23}H_{27}IN_3OOs^+$: m/z 680.1. Found: 680.0. CHN analysis: Found: C, 34.74%; H, 3.26%; N, 5.01%. Calculated for $C_{24}H_{27}F_3IN_3O_4OsS$: C, 34.83%; H, 3.29%; N, 5.08%.

Complex **1**-I·CF_3SO_3 was used in studies of the aqueous chemistry of **1**-I rather than **1**-I·PF_6 because of its improved aqueous solubility. The counter anion has no effect on the anticancer activity or chemical reactivity of the complex.

Synthesis of [Os(η^6-*p*-cym)(5-EtO-AZPY)OH]PF_6 (1-OH·PF_6). To a stirred solution of [Os(η^6-*p*-cym)Cl_2]$_2$ (100 mg, 126.5 µmol) in methanol (3 mL), silver nitrate (58.87 g/L, 1459 µL) in water was added. The mixture turned yellow and a white precipitate formed, which was removed via filtration. A solution of 5-EtO-AZPY (60.4 mg, 265.6 µmol) in methanol (5 mL) was added drop-wise to the yellow solution and it turned brown. The mixture was stirred for 18 h at ambient temperature, then ammonium hexafluorophosphate (206.2 mg, 1.26 mmol) was added. The product was extracted with DCM (10 mL) and washed with water (2 x 10 mL). DCM was removed under reduced pressure and the product was re-dissolved in a minimum amount of methanol (~2 mL), and placed in a freezer overnight. A brown precipitate formed, which was collected via vacuum filtration then washed with ice-cold ethanol (2 x 1 mL), diethyl ether (2 x 5 mL), and dried overnight in a vacuum desiccator. Yield: 160.6 mg (89%). ^1H NMR (D_2O): δ 9.04 (d, 1H, J = 2.2 Hz), 8.76 (d, 1H, J = 9.1 Hz), 7.98-7.95 (m, 3H), 7.76-7.73 (m, 3H), 6.51-6.50 (m, 1H), 6.16-6.15 (m, 1H), 6.01-5.98 (m, 2H), 4.48-4.44 (m, 2H), 2.34 (s, 3H), 2.17 (sept., 1H, J = 6.9 Hz), 1.54 (t, 3H, J = 7.0 Hz), 0.80 (d, 3H, J = 6.9 Hz), 0.68 (d, 3H, J = 6.9 Hz). ESI-MS calculated for $C_{23}H_{28}N_3O_2Os^+$: m/z 570.2. Found: 570.2. CHN analysis: Found: C, 35.54%; H, 3.64%; N, 5.86%. Calculated for $C_{23}H_{28}F_6N_3O_2OsP + CH_2Cl_2$: C, 36.10%; H, 3.79%; N, 5.26%.

Synthesis of [Os(η^6-*p*-cym)(AZPY-NMe$_2$)I]PF$_6$ (2-I·PF$_6$) and [Os(η^6-*p*-cym)(AZPY-NMe$_2$)Cl]PF$_6$ (2-Cl·PF$_6$). These were synthesized and characterised as described previously.[2]

Crystal growth. Crystals of **1**-I (as **1**-I·PF$_6$·0.5EtOH) suitable for x-ray crystallography were grown by dissolving 1-3 mg of solids in ethanol (1 mL) and cooling solutions in a freezer at ca. 253 K. Crystals of **2**-I (as **2**-I·PF$_6$) were grown in a similar manner in methanol.

X-ray crystallography. Diffraction data were collected on an Oxford Diffraction Gemini four-circle system with a Ruby CCD area detector. All structures were refined by full-matrix least squares against F^2 using SHELXL 97 and were solved by direct methods using SHELXS(TREF) with additional light atoms found by Fourier methods. Hydrogen atoms were added at calculated positions and refined using a riding model. Anisotropic displacement parameters were used for all non-H atoms; H-atoms were given an isotropic displacement parameter equal to 1.2 (or 1.5 for methyl and NH H-atoms) times the equivalent isotropic displacement parameter of the atom to which they are attached. The data were processed by the modelling program Mercury 1.4.1.

X-ray crystallographic data for complexes **1**-I·PF$_6$·0.5EtOH and **2**-I·PF$_6$ have been deposited in the Cambridge Crystallographic Data Centre under the accession numbers CCDC 1406692 and 1406691, respectively.

HPLC. The HPLC analysis of complexes was carried out on an Agilent Technologies 1200 series HPLC instrument with a VWD and 100 µL loop, and an Agilent ZORBAX Eclipse Plus C18 250 x 4.6 mm column with a pore size of 5 µm was used. Mobile phases consisted of (A) H_2O + 0.1 %TFA, and (B) MeCN + 0.1 % TFA (HPLC grade materials were used). The following solvent gradient and 1 mL/min flow rate was used.

**Time (min)**	**% (B)**
0	10
30	80
40	80
41	10
55	10

Samples were filtered through Iso-Disc™ filters (PTFE-4-4 4 mm x 0.45 µm). Sample volumes of 50 µM were injected and analysed at a detection wavelength of 254 nm with reference wavelengths set to 360 nm and 510 nm, and additionally, 610 nm with reference wavelength set to 360 nm for **2**-I and **2**-Cl. The chromatograms were analysed using ChemStation software and peaks with intensity >10 mAU were integrated. Chromatograms were generated using MS Excel 2010. For reactions labelled as '0 h' reaction time, samples were injected within 5 min of mixing.

Radio-HPLC. An Agilent 1200 series instrument with a VWD, a 200 µL loop, and an Agilent ZORBAX Eclipse Plus C18 250 x 4.6 mm column with a pore size of 5 µm was used. The HPLC was fitted with either a raytest Gabi Star gamma detector with a raytest NaI-detector 1x1`` Std gamma probe, or a LabLogic FLOW-COUNT gamma detector with a LabLogic B-FC-3200 gamma probe. The same mobile phase and solvent gradient was used as previously described with a 1 mL/min flow rate. Unfiltered volumes of <200 µL were injected and analysed at a detection wavelength of 254 nm. HPLC chromatograms were analysed and integrated using GINA Star 5.8 or Laura-HPLC 4.0.2.75 software.

LC-MS. A Bruker Amazon X+ instrument coupled with an Agilent Technologies 1200 series HPLC instrument was used. The same HPLC column, method and conditions were as described above. Sample injection volumes were 20 µL and the mass spectrometer was operated in electrospray positive mode with a scan range of 50-2000 m/z.

Radio-TLC. Riedel-de Haën TLC-sheets (12 x 100 mm, silica gel 60 F254, layer thickness 0.2 mm) were used. Plates were spotted with 3 µL of reaction mixture at the baseline, then placed inside sealed plastic tubes with eluent water:methanol (1:1 v/v, 700 µL). The solvent front was allowed to travel 90 mm from the baseline. Plates were scanned on a LabLogic MINI-SCAN TLC scanner with a LabLogic B-FC-3200 gamma probe at a scan rate of 0.25 mm/s. TLC chromatograms were analysed and integrated using Laura-TLC version 4.0.2.75 software.

Blood serum stability assays. Human blood serum (900 µL) was combined with radio-tracer complex (100 µL, ~500 KBq). Samples were incubated at 310 K with 300 rpm stirring and analyzed after 1 h and 24 h by radio-HPLC. The serum samples were

treated by combining with 1 part MeCN and centrifuging for 5 min (>10000 rpm) to remove serum proteins before radio-HPLC analysis.

Cell culture. MCF-7 breast cancer cells and A2780 ovarian cancer cells were purchased from the European Collection of Cell Cultures and used between 5 – 25 passages. Cells were grown as adherent monolayers in either Dulbecco's modified Eagle's medium (D-MEM, for MCF-7 cells) or Rosswell Park Memorial Institute medium 1640 (RPMI-1640, for A2780 cells) supplemented with 10% v/v foetal calf serum, 1% v/v 2 mM *L*-glutamine, and 1% v/v penicillin/streptomycin mixture. At KCL all cell counting was carried out using an Invitrogen countess automated cell counter.

***In vitro* stability assays.** MCF-7 cells were seeded in a 24-well plate using $1x10^6$ per well. Cells were pre-incubated in drug free medium for 24 h at 310 K in a 5% CO_2 humidified atmosphere. The medium was removed and fresh medium was added; 900 µL of medium combined with 100 µL radiotracer (~500 kBq). A second 24-well plate was prepared for control experiments containing no cells. Both well plates were incubated for 24 h and supernatants from wells were removed at different time points: 2, 4, 8 and 24 h. The supernatants were combined with 1 part MeCN and centrifuged for 5 min (>10000 rpm) to remove cell culture proteins/dead cells before radio-HPLC analysis.

Cellular accumulation experiments. $1x10^6$ MCF-7 cells were seeded into borosilicate glass culture tubes (12 x 75 mm) with 1 mL cell culture medium, and combined with the tracer (30 µL, 15-17 KBq). Both the cells and control samples containing no cells were incubated at 310 K for pre-determined time periods in triplicate. The tubes were cooled in ice/water, centrifuged at 1000 rpm for 3 min, and the supernatants were removed and collected. The cells were washed with ice-cold PBS solution (2x) and the washings were combined with the supernatant fractions. Cell pellets were digested in 1 M NaOH solution (1 mL) and collected separately. The tubes were washed with PBS solution (2x) and residues were combined with digested cell fractions. Control experiments were treated in the same way. Cellular uptake of ^{131}I was measured at various time points: 5, 10, 30, 60, 180, 360 min. The number of counts per minute (CPM) of the supernatants and digested cell fractions were measured in a LKB Wallac 1282 Compugamma universal gamma counter, using EdenTerm V1.21 software. The cellular protein content was determined for digested

cells using a calibration curve (Fig. S6). The data were processed using MS excel and standard deviations were calculated.

***In vitro* growth inhibition assay.** Approximately 5000 A2780 or MCF-7 cells were seeded per well in 96-well plates. The cells were pre-incubated in drug-free media at 310 K for 48 h before adding various concentrations of the osmium complex. Cells were exposed to complexes for 24 h at 310 K. The supernatants were removed by suction and each well was washed with PBS. The cells were allowed to recover for 72 h in a drug-free medium at 310 K. The SRB assay was used to determine cell viability. Absorbance measurements of the solubilised dye (BioRad iMark microplate reader, 470 nm filter) allowed the determination of viable treated cells compared to untreated controls. IC_{50} values (the concentration at which 50% cell death occurs) were determined as triplicates of duplicates for each complex. ICP-MS was used to determine the osmium concentrations of the initial stock solutions of the complexes.

Hydrolysis in presence of GSH. Samples of **1**-OH (1 mM) were prepared in phosphate buffer (100 mM, pH 7.4) with GSH (1 mol. equiv.), or no GSH (control study). Samples were incubated at 310 K for 0 and 24 h, then stored in a freezer. The samples required x10 dilution in water before HPLC analysis was carried out. The samples that received 24 h incubation were later analysed via LC-MS to identify the species in solution.

Likewise, complexes **1**-I, **2**-I and **2**-Cl (75 µM) were prepared in phosphate buffer solution (75 mM, pH 7.4; with or without 25 mM NaCl) with different quantities of GSH (1 to 100 mol. equiv.). Samples were incubated for different time periods (0 to 24 h) and did not require dilution prior to HPLC or LC-MS analysis. The HPLC wavelengths of detection used were 254 nm (referenced to 510 nm) for **1**-OH and **1**-I, and 610 nm (referenced to 360 nm) for **2**-I and **2**-Cl.

Hydrolysis in presence of ascorbic acid. Complex **2**-I (30 µM) was prepared with ascorbic acid (3.33 mol. equiv.) in phosphate buffer solution (75 mM, pH 7.3), and under a N_2 atmosphere. Samples were incubated at 310 K for 0, 1, 3, 6 and 24 h then stored in a freezer. The samples were then analysed by HPLC. The wavelength of detection observed was 610 nm (referenced to 360 nm). Control samples were also prepared containing no ascorbic acid and were incubated for 0 and 24 h.

EPR. EPR spectra were recorded at ambient temperature on a Bruker EMX (X-band) spectrometer fitted with a cylindrical Tm110 mode cavity (Bruker 4103TM). Samples were contained in quartz capillary tubes (I.D. 1.0 mm; O.D. 1.2 mm; Wilmad Labglass) sealed with T-Blu Tac®, and placed inside larger quartz tubes (O.D. 2.0 mm) to achieve easy and accurate positioning of the sample inside the resonator. Typical key EPR spectrometer settings were: modulation amplitude 2.0 G, microwave power 0.63 mW, $1.0x\ 10^5$ receiver gain, conversion time 81.92 ms, time constant 81.92 ms, sweep width 200 G, and a repeated number of 10 X-scans with a resolution in Y of 5 or 9. Solutions of complexes (1 mM) were prepared in phosphate buffer solution (75 mM) with DEPMPO (6 mM) and H_2O_2 (10 mM). For complexes **1**-I and **1**-Cl, DMF (50%) was used to solubilise the complexes to 1 mM. The EPR spectra were recorded immediately after sample preparation. EPR spectra were analysed and simulated using the EASYSPIN software.[3]

pK_a determination. A 2 mM solution of complex **1**-OH was prepared in D_2O, and 4 mM dioxane was added as an internal NMR standard (at 3.75 ppm). The solution was aliquoted into 8 samples of 600 μL and the pH* values were adjusted sequentially by adding 1-10 μL of either KOD or $DClO_4$ (0.01, 0.1, 1, 2, 3, 4, 6, 8 or 10 M) in D_2O. The pH* values were measured over a range of 1.5 – 13.5 using a pH meter (without correction for the effect of deuterium on the glass electrode). Changes in the chemical shifts of the peaks of complex **1**-OH were followed by ^1H NMR. The pH* values were corrected using the equation; pH = 0.929pH* + 0.41,[4] and the data were fitted to the Henderson-Hasselbalch equation using Origin 8.5.

References

1. J. Tönnemann, J. Risse, Z. Grote, R. Scopelliti, K. Severin, *Eur. J. Inorg. Chem.* **2013**, 4558-4562.
2. Y. Fu, A. Habtemariam, A. M. Pizarro, S. H. van Rijt, D. J. Healey, P. A. Cooper, S. D. Shnyder, G. J. Clarkson, P. J. Sadler, *J. Med. Chem.* **2010**, *53*, 8192-8196.
3. S. Stoll, A. Schweiger, *J. Magn. Reson.* **2006**, *178*, 42-55.
4. A. Krężel, W. Bal, *J. Inorg. Biochem.* **2004**, *98*, 161-166.

Table S1. Species identified by HPLC/LC-MS and their molecular formulae, calculated and observed m/z values, and HPLC retention times for reactions between **1-**I and **2-**I with GSH.

Species	Molecular formula	Calculated m/z	Observed m/z	HPLC retention time (min)[a]
**1-I**	$C_{23}H_{27}IN_3OOs^+$	680.08	680.00	24.05
**1-OH**	$C_{23}H_{28}N_3O_2Os^+$	570.18	570.11	12.61
**1-SG**	$C_{33}H_{43}N_6O_7OsS^+$	859.25	859.16	13.38
**1-SG**(+H⁺)	$C_{33}H_{44}N_6O_7OsS^{2+}$	430.13	438.07	
**1-SOG**	$C_{33}H_{43}N_6O_8OsS^+$	875.25	875.14	11.68
**1-SOG**(+H⁺)	$C_{33}H_{44}N_6O_8OsS^{2+}$	438.13	438.07	
**2-I**	$C_{23}H_{28}IN_4Os^+$	679.10	679.02	23.02
**2-Cl**	$C_{23}H_{28}ClN_4Os^+$	587.16	587.21	19.67
**2-OH**	$C_{23}H_{29}N_4OOs^+$	569.20	569.17	12.53
**2-SG**	$C_{33}H_{44}N_7O_6OsS^+$	858.27	858.19	13.29
**2-SG** (+H⁺)	$C_{33}H_{45}N_7O_6OsS^{2+}$	429.64	429.65	
**2-SOG**	$C_{33}H_{44}N_7O_7OsS^+$	874.26	874.15	11.54
**2-SOG** (+H⁺)	$C_{33}H_{45}N_7O_7OsS^{2+}$	437.64	437.64	

[a] The retention times listed here were determined on a different HPLC column from that used in the main script.

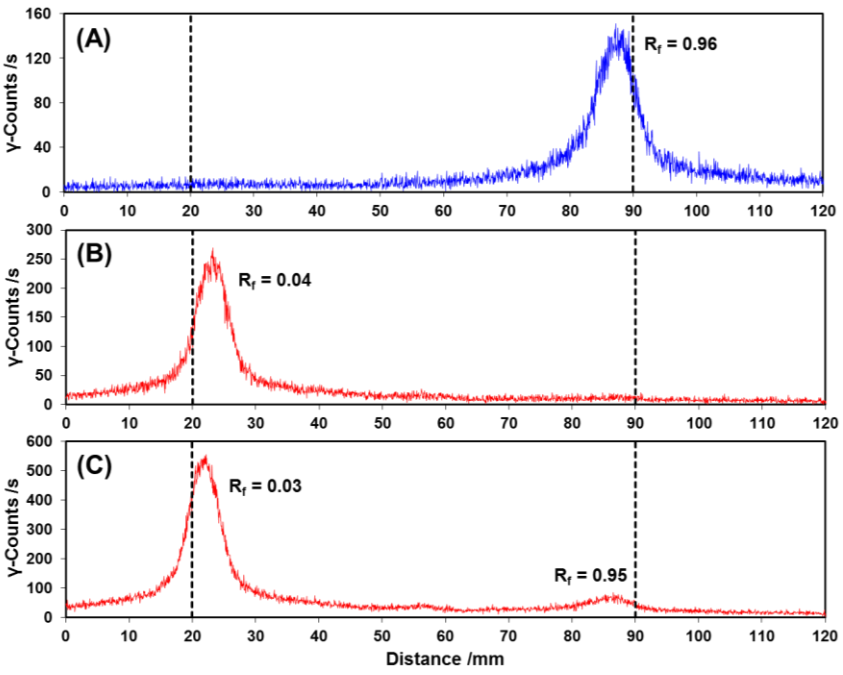

Figure S1. Radio-TLC analysis of (A) aqueous NaI-131, (B) the radio-synthesis of **1**-[^{131}I] after 2 h, (C) the radio-synthesis of **2**-[^{131}I] after 2 h. The decrease in R_f signifies the transformation from free $^{131}I^-$ to complex-bound ^{131}I.

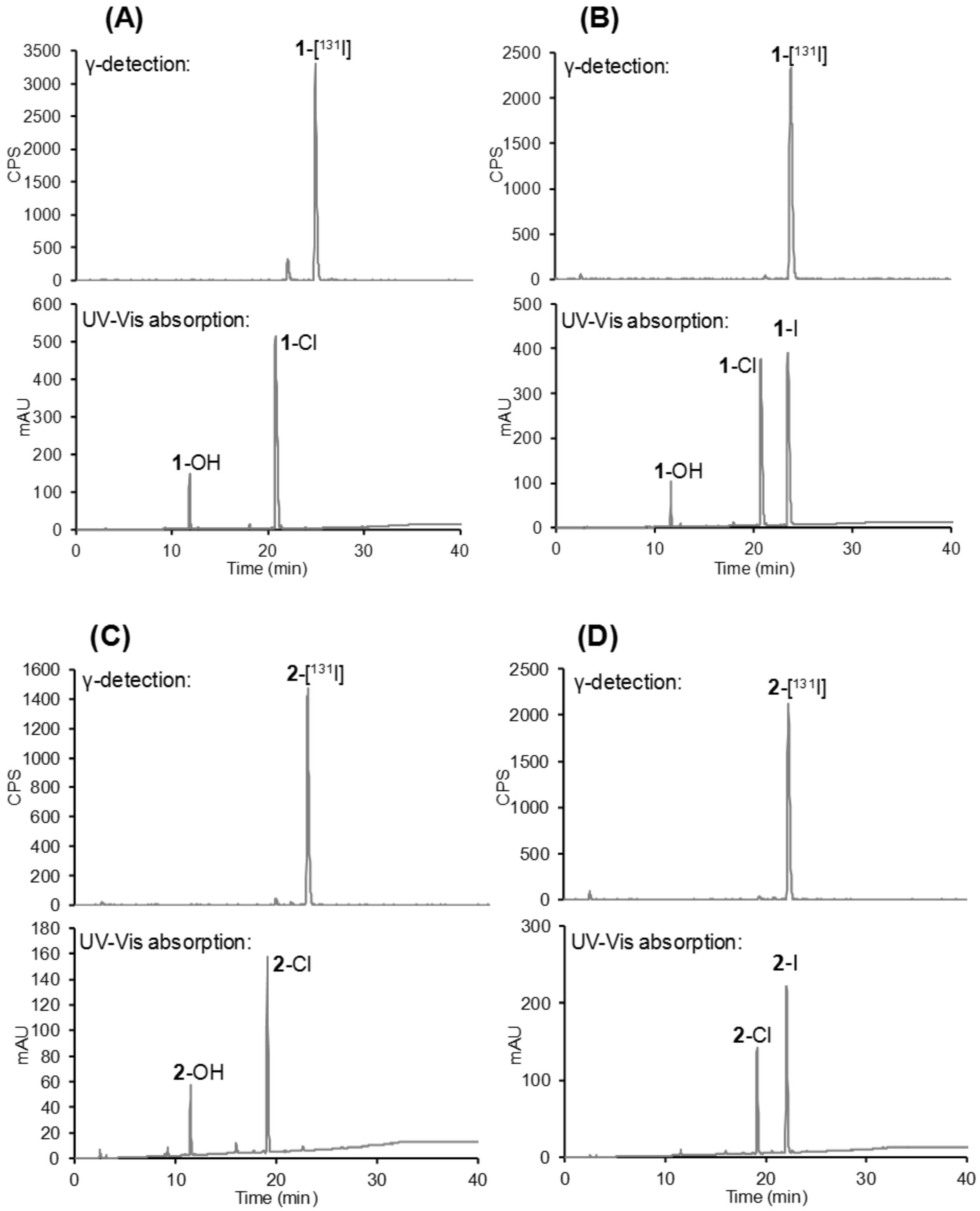

Figure S2. Radio-HPLC chromatograms of reaction mixtures from the synthesis of radio-labelled ^{131}I-complexes, showing the major species present. (A); Synthesis of **1**-[^{131}I]. (B) Synthesis of **1**-[^{131}I] in the presence of 0.5 mol. equiv. NaI. (C); Synthesis of **2**-[^{131}I]. (D); Synthesis of **2**-[^{131}I] in the presence of 0.5 mol. equiv. NaI.

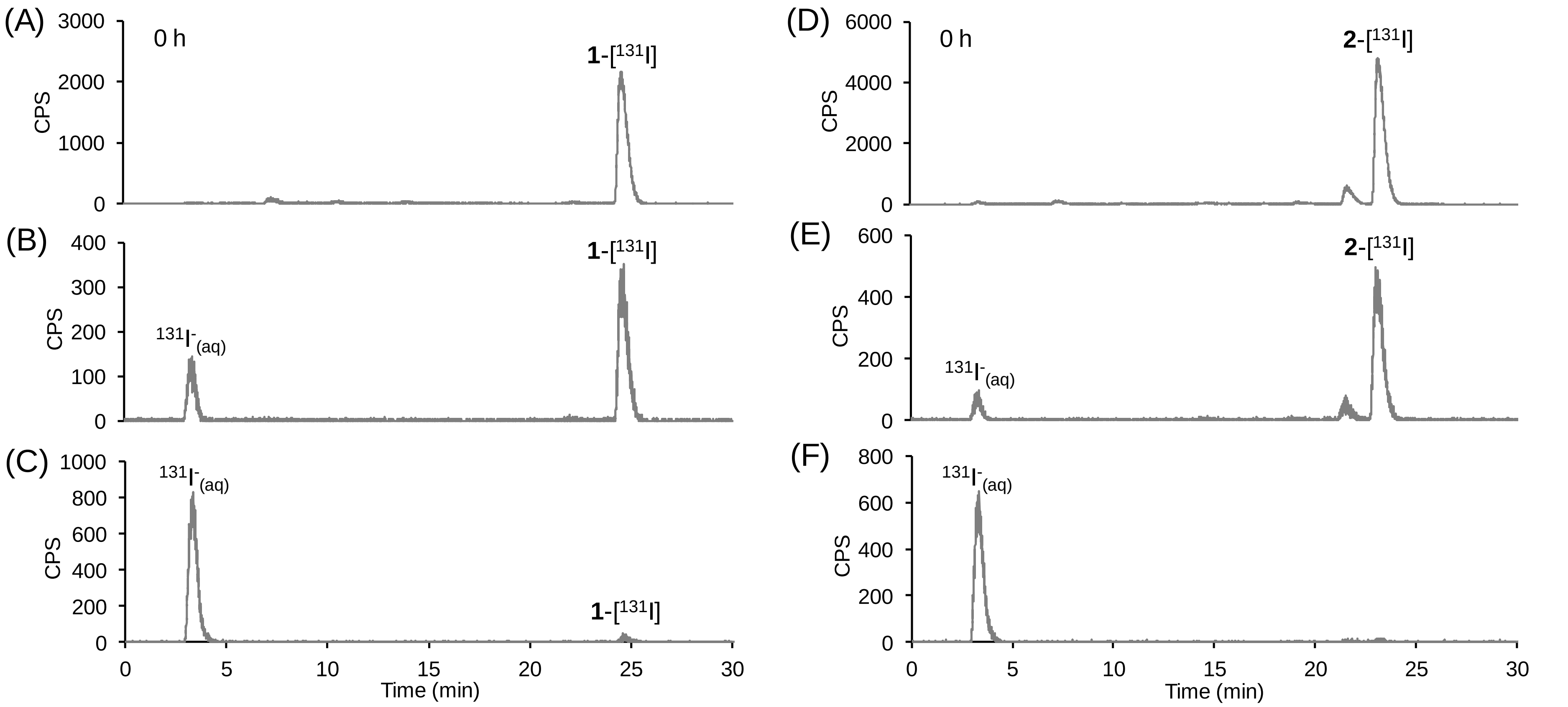

Figure S3. Radio-HPLC chromatograms for studies on the stabilities of **1**-[^{131}I] and **2**-[^{131}I]. (A) **1**-[^{131}I] before incubation. (B) **1**-[^{131}I] after incubation in human blood serum. (C) **1**-[^{131}I] after incubation in cell culture medium in the presence of MCF-7 cells. (D) **2**-[^{131}I] before incubation. (E) **2**-[^{131}I] after incubation in human blood serum. (F) **2**-[^{131}I] after incubation in cell culture medium. Incubations were carried out at 310 K for 24 h.

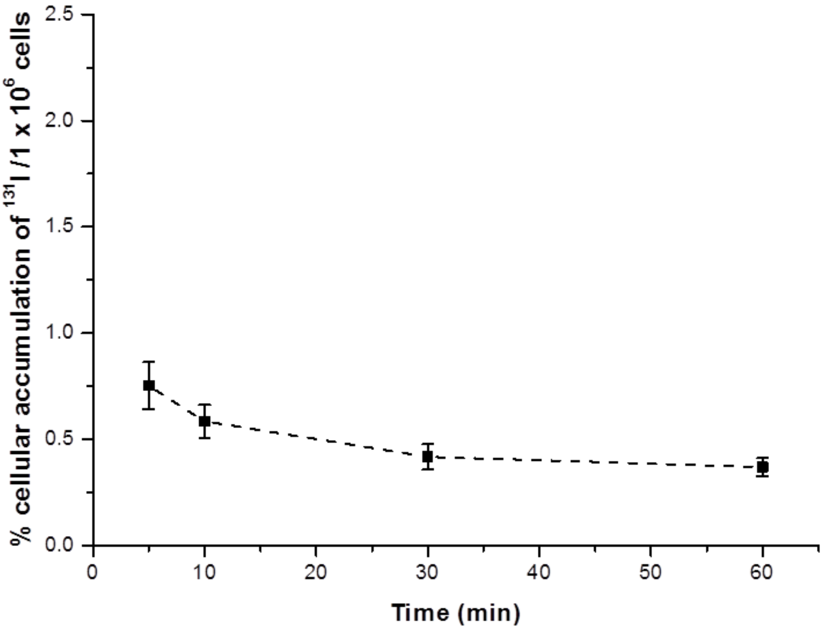

Figure S4. Cellular accumulation of iodine-131 in MCF-7 cells at various times after incubation with **1**-[^{131}I].

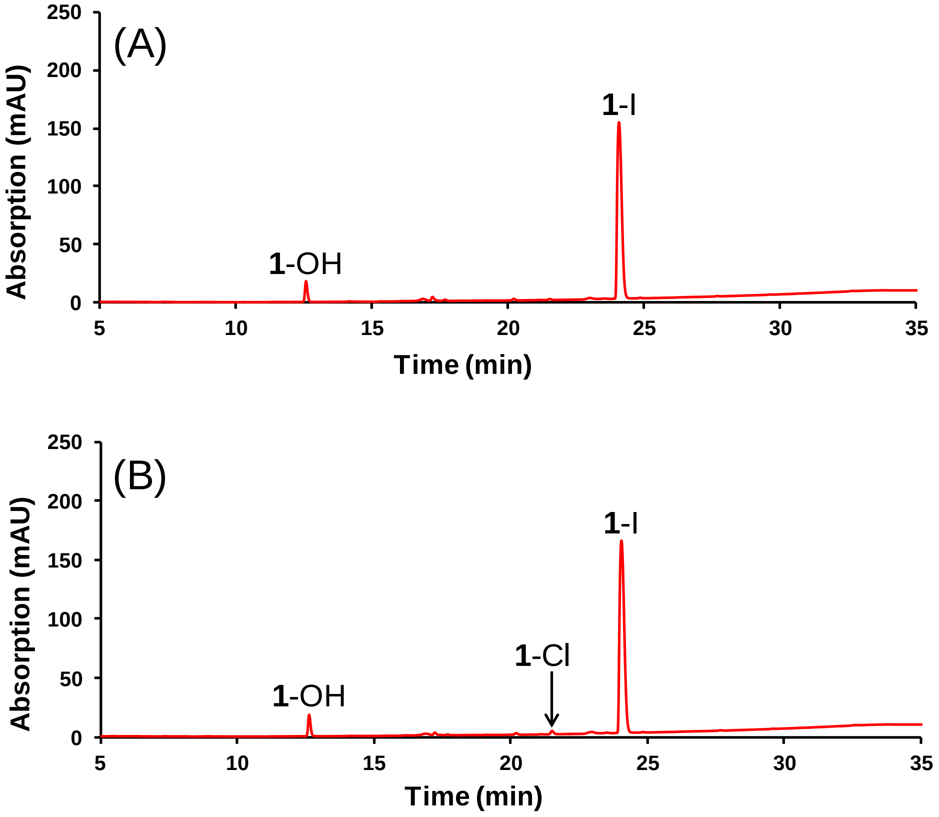

Figure S5. HPLC chromatograms of complex **1**-I after incubation in phosphate buffered saline (pH 7.4) at 310 K for 24 h. (A) intranuclear conditions; 4 mM NaCl. (B) cytoplasmic conditions; 23 mM NaCl.

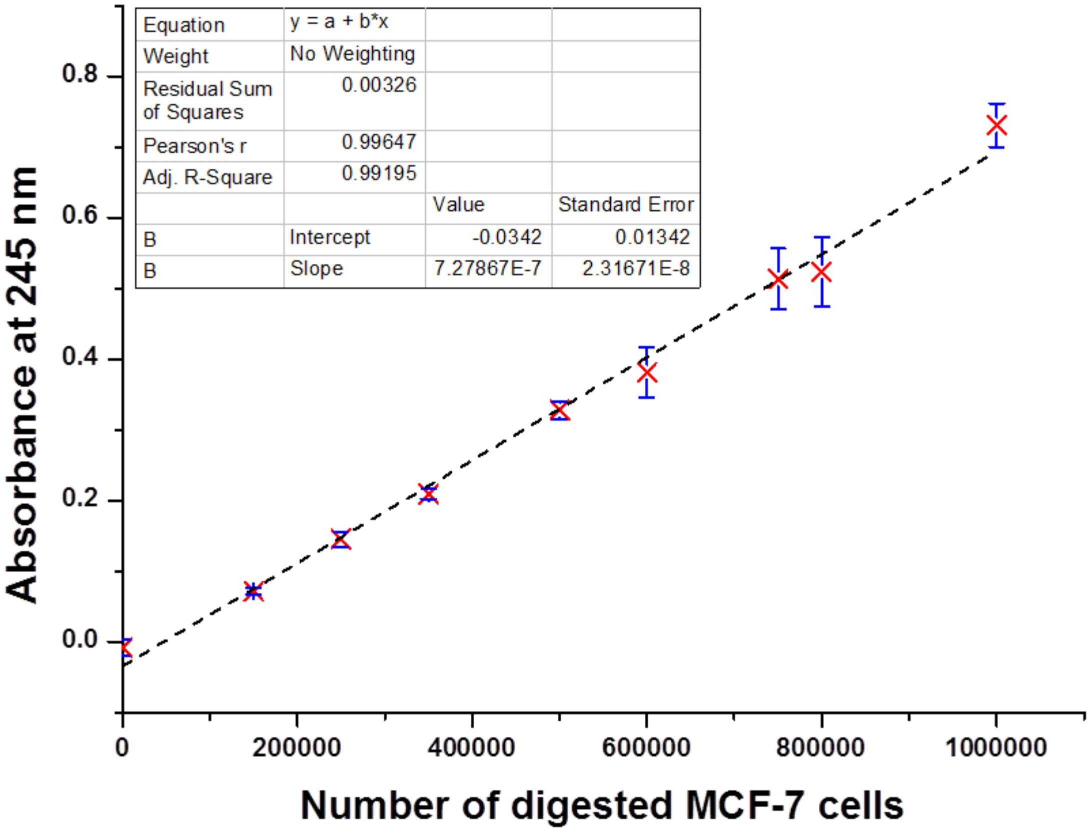

Figure S6. Calibration curve for UV absorbance at 245 nm against number of digested MCF-7 cells. (See cell accumulation experiments protocol, page S7).

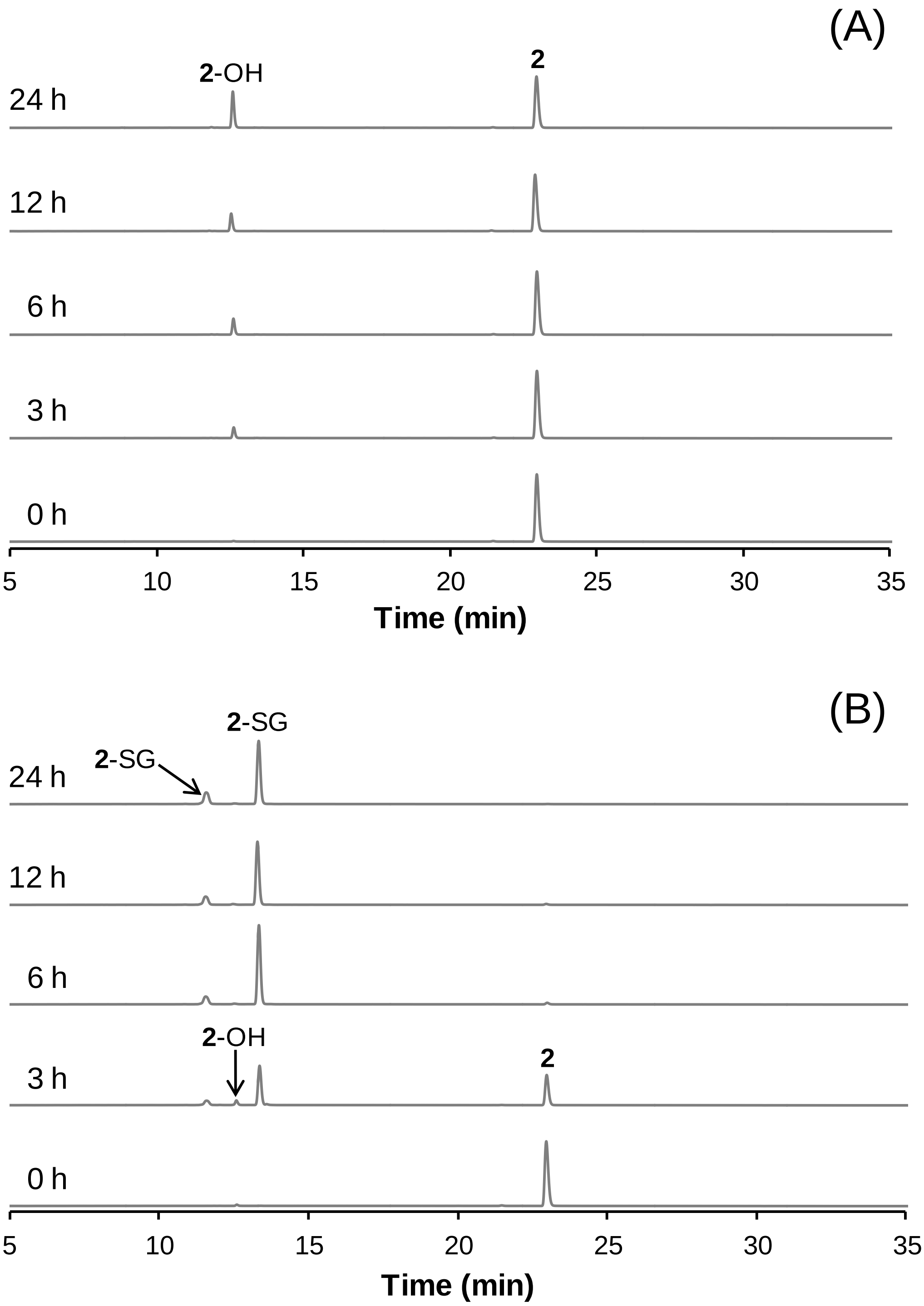

Figure S7. HPLC chromatograms for complex **2**-I after incubation with GSH at 310 K in phosphate buffer (75 mM, pH 7.4) for various times. (A) 75 μM **2**-I with 1 mol. equiv. GSH. (B) 75 μM **2**-I with 100 mol. equiv. GSH.

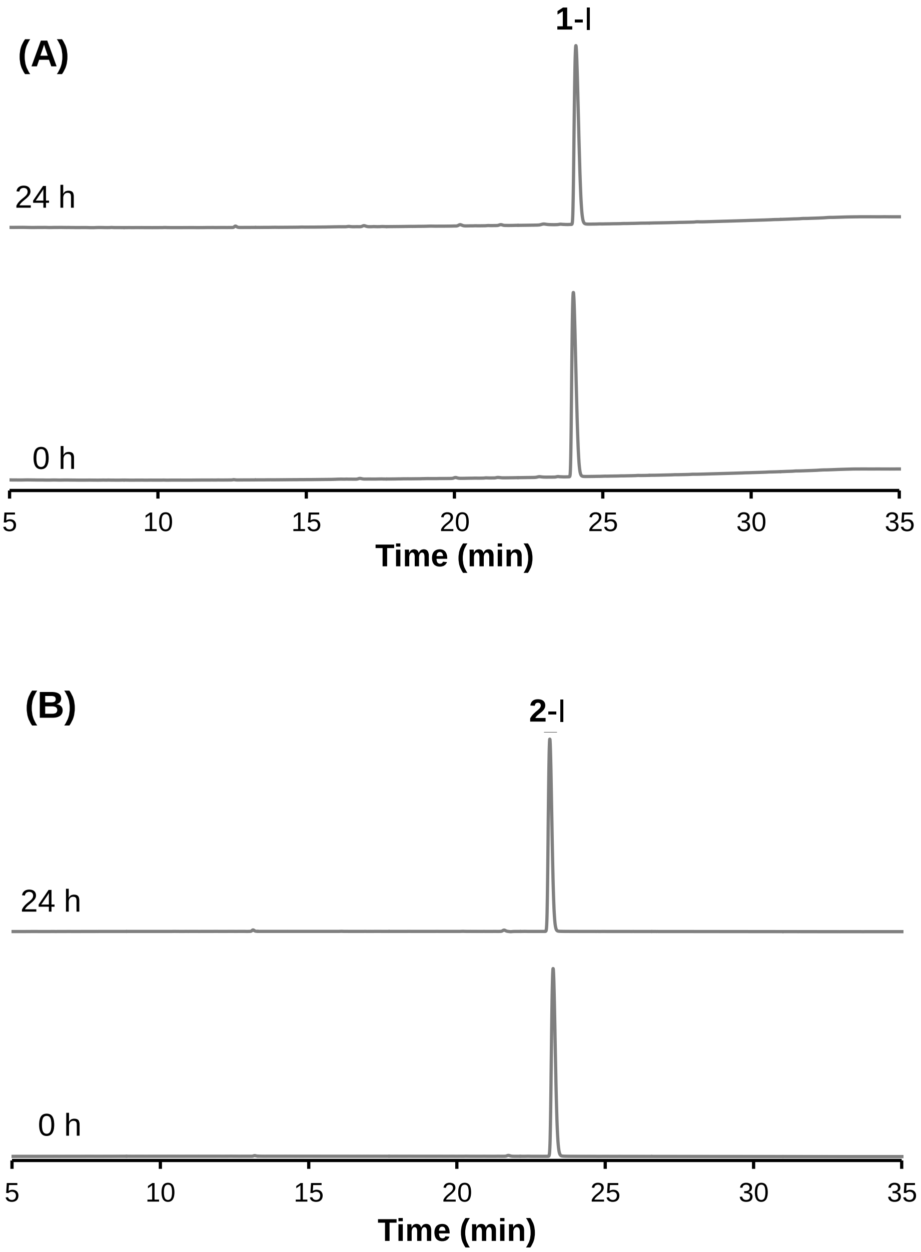

Figure S8. HPLC chromatograms for control experiments. Complexes (75 μM) were incubated in phosphate buffer (75 mM, pH 7.4) at 310 K for 24 h. (A) complex **1-I**. (B) complex **2-I**.

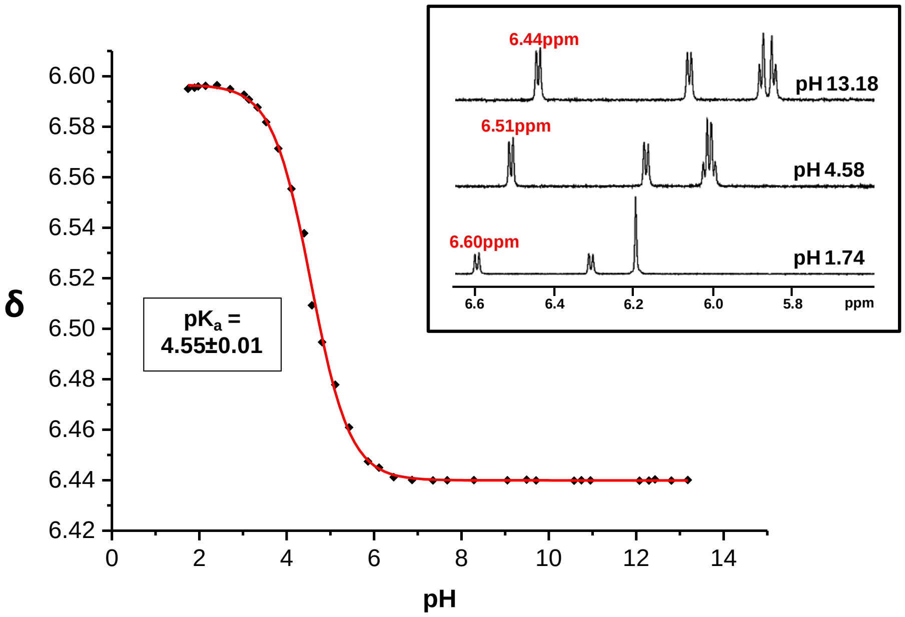

Figure S9. Variation of the chemical shift of an aromatic *p*-cym proton of **1**-OH with pH. The red line represents a computer best fit to the Henderson-Hasselbalch equation.

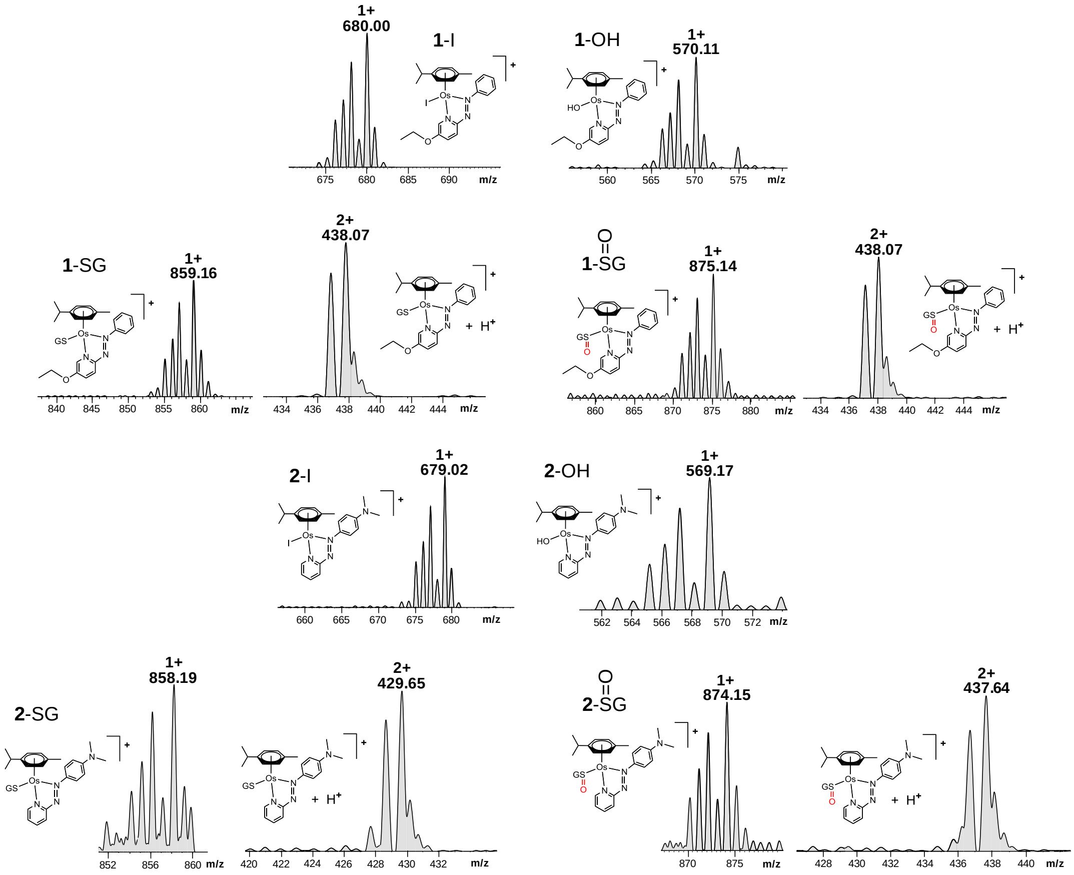

Figure S10. Mass spectra of all the species detected in HPLC chromatograms, analysed via LC-MS.

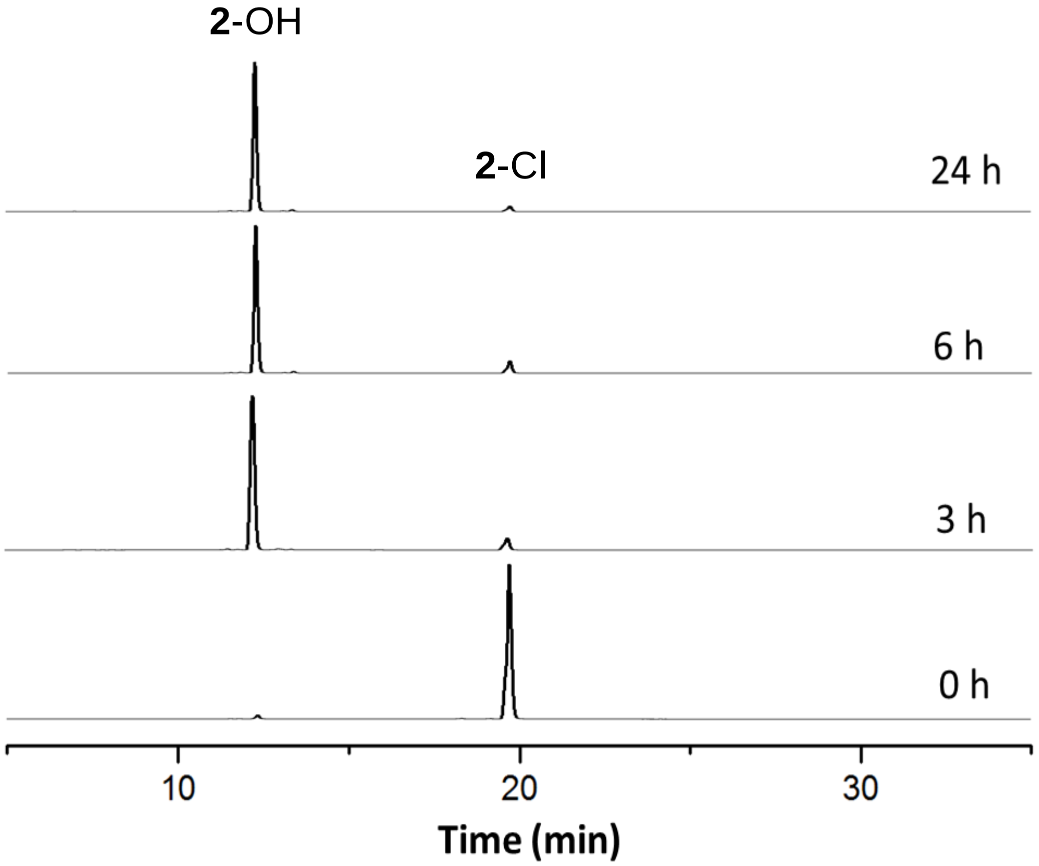

Figure S11. HPLC chromatograms for complex **2**-Cl (75 μM) after incubation with GSH (1 mol. equiv.) at 310 K in phosphate buffer (75 mM, pH 7.4) for various times.

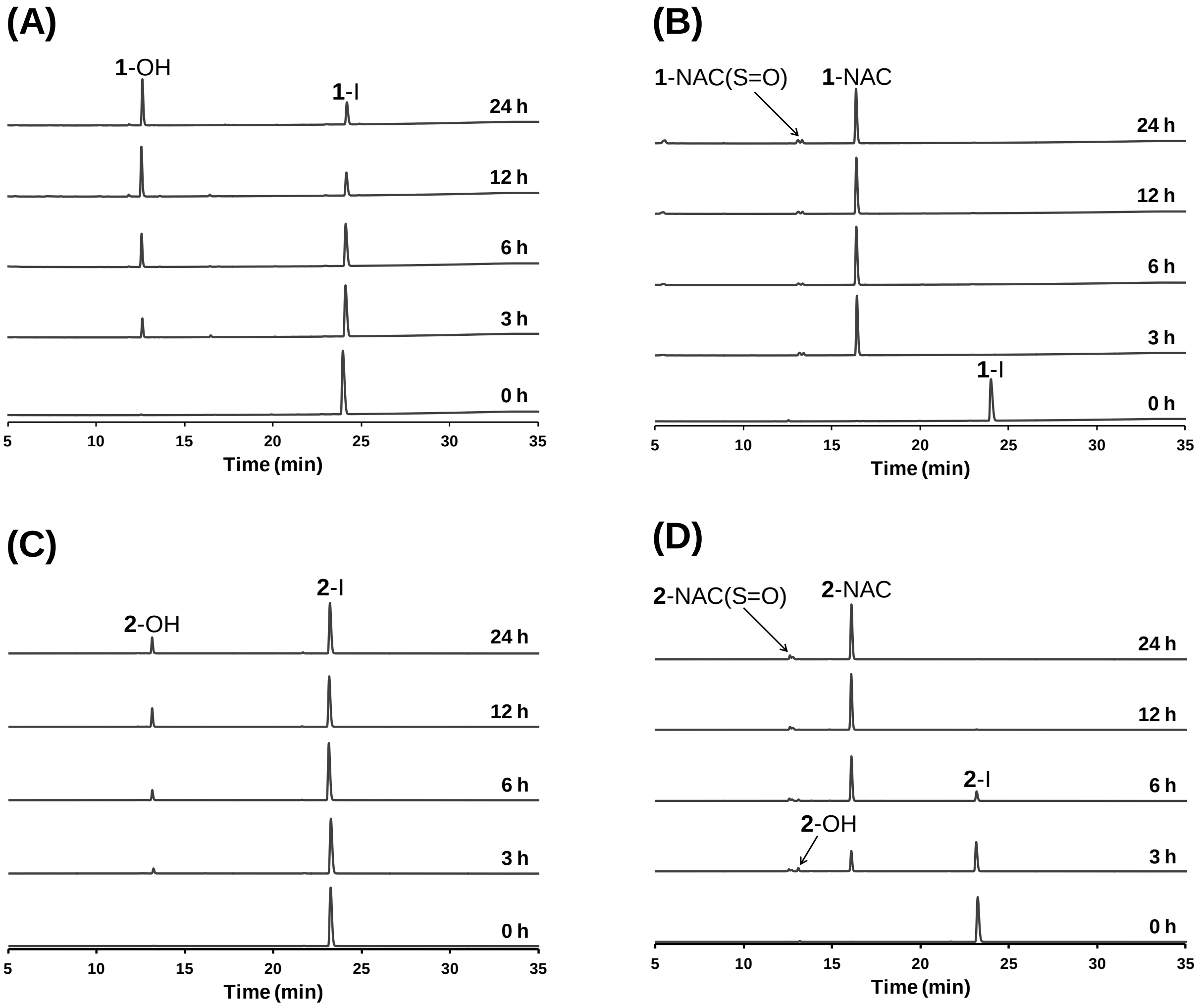

Figure S12. HPLC chromatograms for complexes **1**-I and **2**-I after incubation with N-acetyl-*L*-cysteine (NAC) at 310 K in phosphate buffer (75 mM, pH 7.4). (A) 75 μM **1**-I with 1 mol. equiv. NAC. (B) 75 μM **1-I** with 100 mol. equiv. NAC. (C) 75 μM **2**-I with 1 mol. equiv. NAC. (D) 75 μM **2**-I with 100 mol. equiv. NAC.

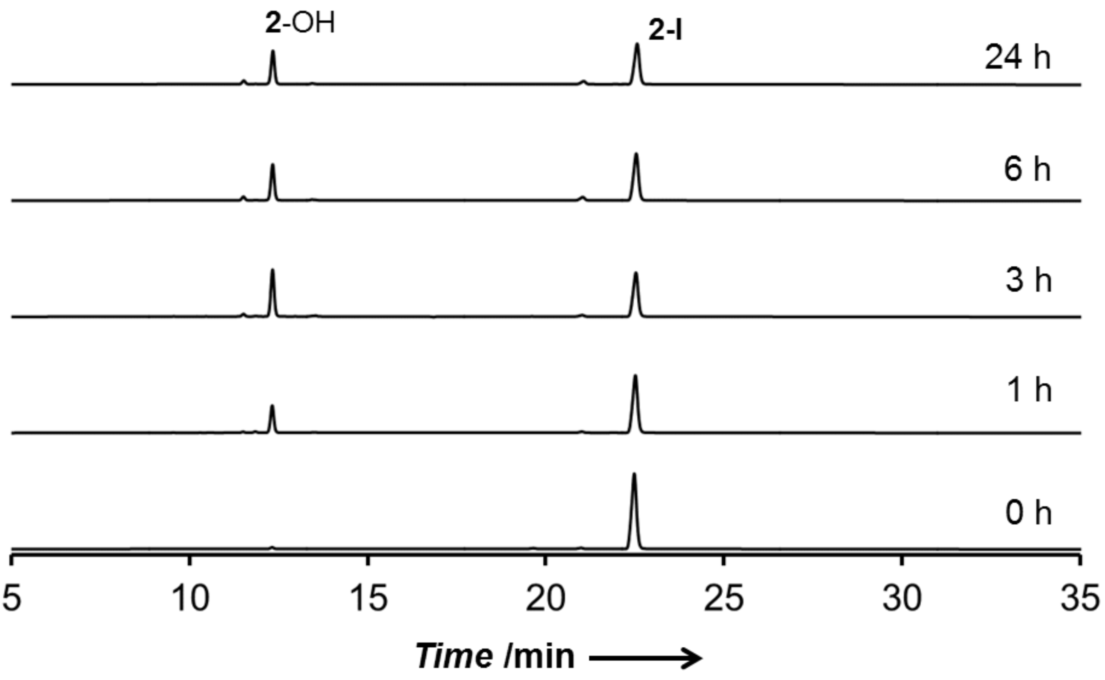

Figure S13. HPLC chromatograms for complex **2**-I (30 μM) after incubation with 3.33 mol. equiv. ascorbic acid at 310 K in phosphate buffer (75 mM, pH 7.4) for various times.

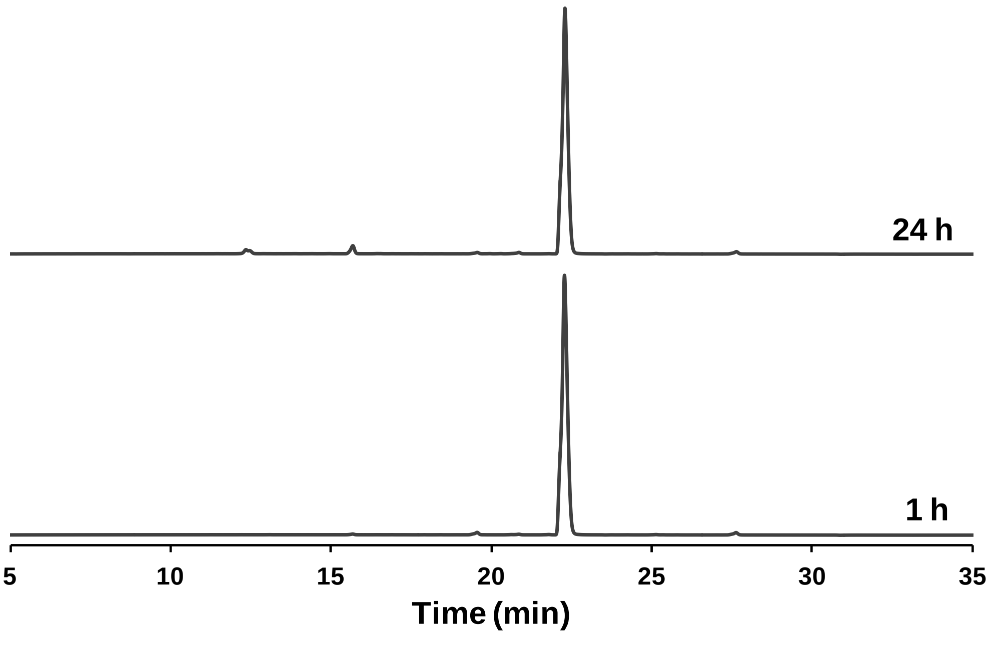

Figure S14. HPLC chromatograms of complex **2**-I (100 μM) after incubation with 100 mol. equiv. NAC at 310 K in phosphate buffer (75 mM, pH 7.4), containing 30% v/v acetone.

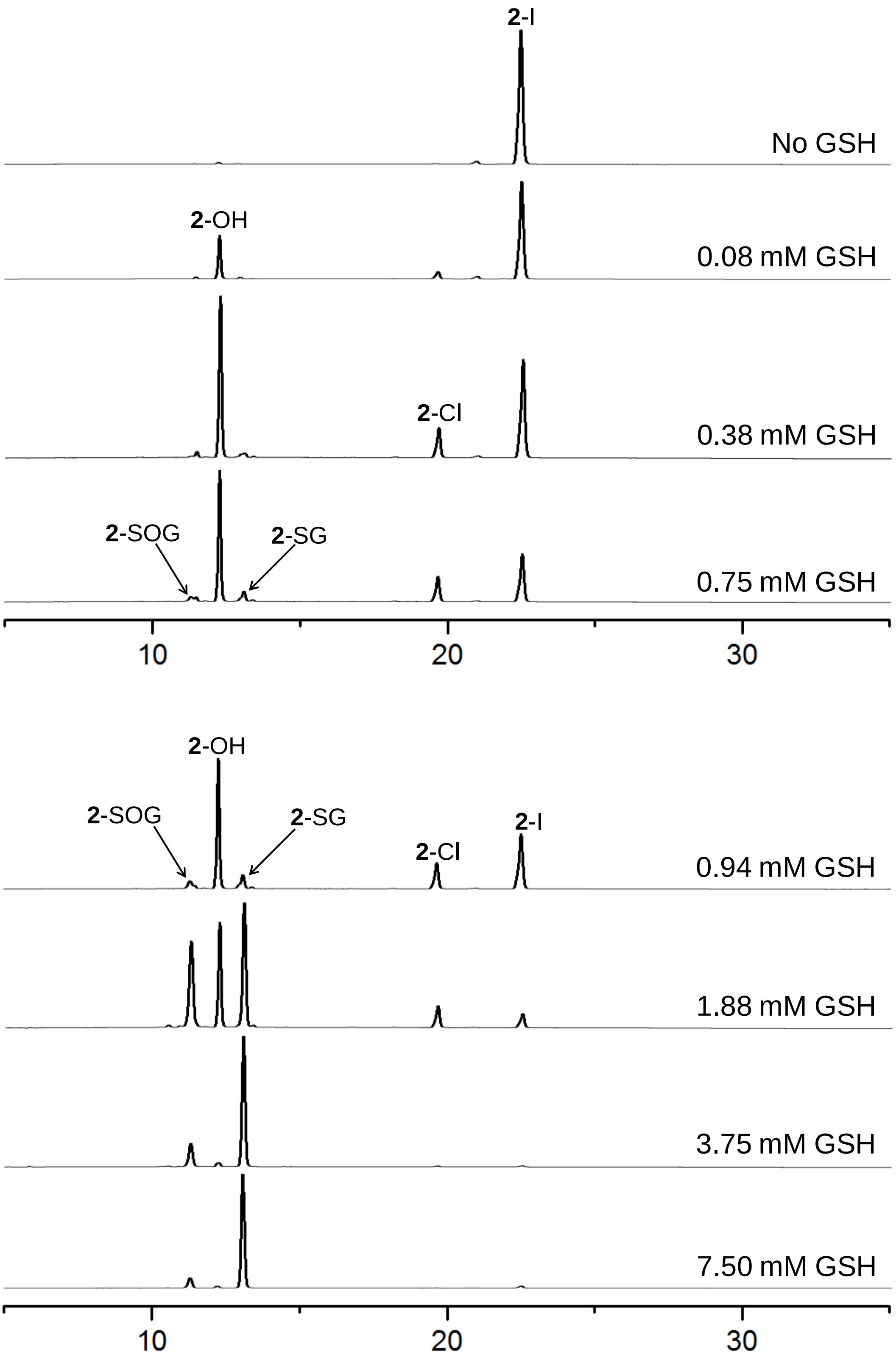

Figure S15. HPLC chromatograms of complex **2**-I (75 µM) after 24 h incubation at 310 K in phosphate buffer (75 mM, pH 7.4) and NaCl (25 mM), with various concentrations of GSH (0 - 7.5 mM).

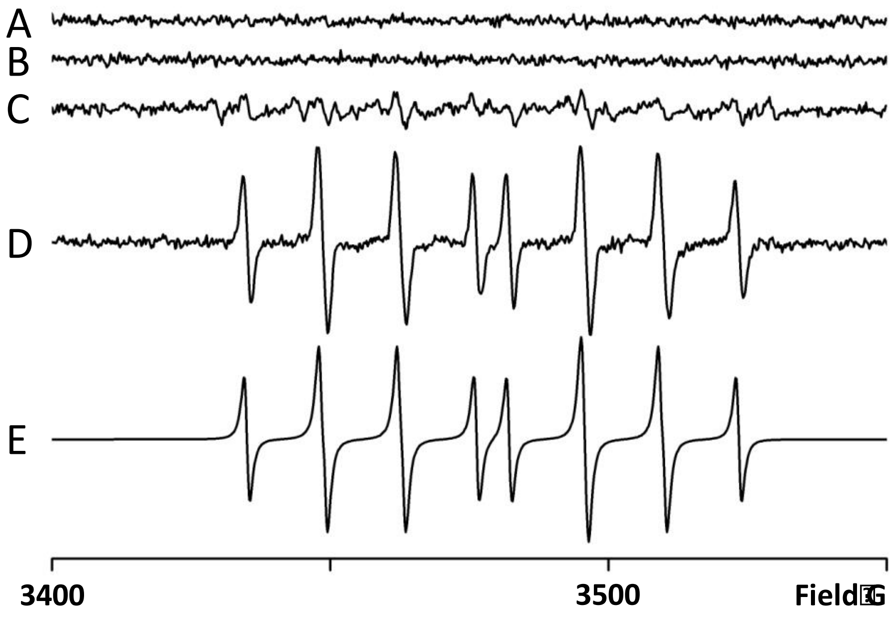

Figure S16. X-band EPR spectra for the reaction between 1 mM **1**-OH, 6 mM DEPMPO, and 10 mM H_2O_2 in 75 mM phosphate buffer, showing the trapping of HO· radicals and quenching by ethanol (10 mM). (A) H_2O_2 + DEPMPO; (B) **1**-OH + DEPMPO; (C) **1**-OH + H_2O_2 + DEPMPO + ethanol; (D) **1**-OH + H_2O_2 + DEPMPO; (E) Simulated spectrum using the parameters in Fig 5 caption.

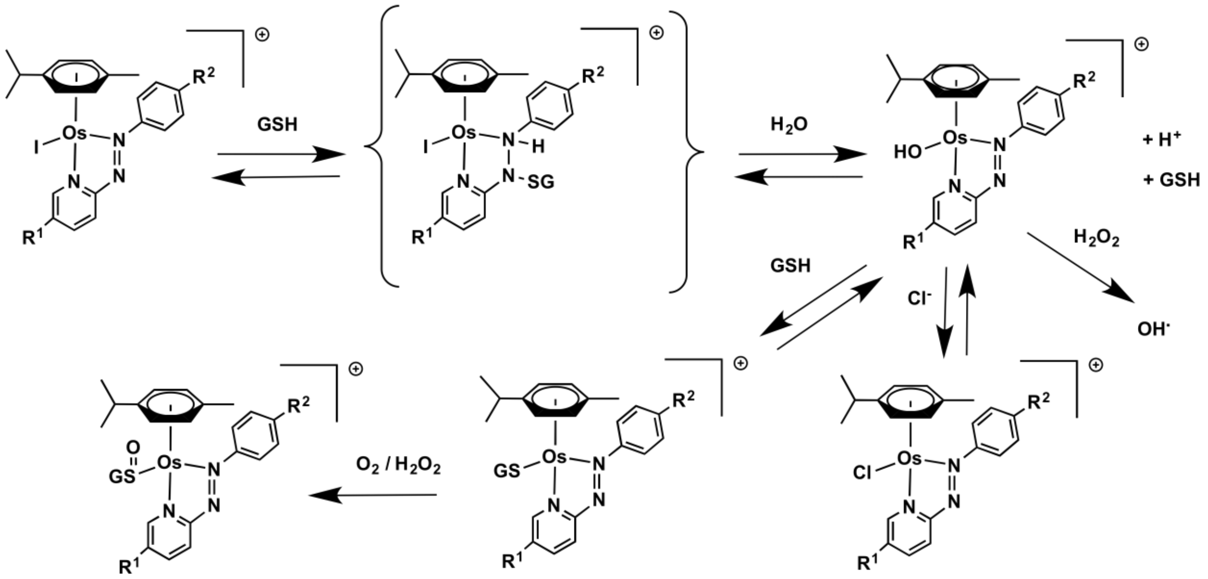

Figure S17. Some possible pathways for the activation of iodido Os^{II} arene azopyridine complexes by reaction with glutathione.
